# Utility of stool culture for the diagnosis and management of paediatric TB in a low-burden setting

**DOI:** 10.5588/ijtldopen.25.0794

**Published:** 2026-05-11

**Authors:** R.E. Elphinstone, S.L. Silverberg, M. Richard-Greenblatt, R. Lam, V. Waters, I. Kitai

**Affiliations:** 1 Division of Infectious Diseases, Department of Paediatrics, The Hospital for Sick Children, Toronto, ON, Canada;; 2 Edwin SH Leong Centre for Healthy Children, University of Toronto, Toronto, ON, Canada;; 3 Department of Paediatric Laboratory Medicine, The Hospital for Sick Children, Toronto, ON, Canada;; 4 Department of Laboratory Medicine and Pathobiology, University of Toronto, ON, Canada;; 5 Lawrence Bloomberg School of Nursing, University of Toronto, ON, Canada;; 6 Department of Paediatrics, University of Toronto, Toronto, ON, Canada.

**Keywords:** tuberculosis, child TB, time to positivity, DST, Canada

Dear Editor,

Microbiologic confirmation of TB disease is important for obtaining drug susceptibility tests (DSTs), allowing appropriate management of drug-resistant TB or when first-line agents cause side-effects.^1^ Obtaining a positive culture in paediatric cases is difficult: disease is often paucibacillary, children may not effectively expectorate sputum, and extra-pulmonary disease is common.^2^ While molecular tests on stool are endorsed by the WHO for diagnosing TB disease in young children,^3,4^ there are very few data about the utility of stool culture for *Mycobacterium tuberculosis* complex (MTBC) in paediatric TB. In Ontario, Canada, the reference mycobacteriology laboratory cultures stool specimens for *M. tuberculosis* but due to high volumes only performs MTBC polymerase chain reaction (PCR) on first-time acid-fast bacilli (AFB) smear-positive specimens. We report on two cases where stool cultures were helpful in patient management and our subsequent experience of routine stool culture in consecutive cases hospitalised for TB workup at SickKids, the regional referral centre that manages most paediatric TB in the Greater Toronto Area.

Case 1: An 11-year-old Canadian-born child with Leber’s optic neuropathy developed intestinal obstruction and underwent resection of an abdominal mass during a visit to Pakistan. Histology of the specimen suggested TB; no microbiologic testing was done. The family returned to Canada before drug treatment with ongoing fever and weight loss. Colonoscopy-derived biopsies of the ileum and colon were culture-negative for MTBC. Biopsy of intraabdominal lymph nodes was deemed to be difficult with low yield. However, one of three stool specimens collected at admission grew MTBC susceptible to all first-line agents after 28 days. Ethambutol was therefore avoided in this child with visual impairment. The patient was treated for 10 months with initial first-line treatment of isoniazid, rifampin, and pyrazinamide with resolution of symptoms and clinical cure.

Case 2: A 14-year-old with intrathoracic and supraclavicular lymphadenopathy and terminal ileal thickening with negative sputum cultures underwent an interventional-radiology-guided biopsy of a supraclavicular node that was non diagnostic and later underwent an excisional biopsy of the node. Two days after this biopsy a stool specimen taken 18 days previously grew MTBC. Resulting DSTs were useful as the patient rapidly developed hepatotoxicity on initial treatment.

Following these cases, we recommended stool culture as part of the TB workup for admitted patients at our institution. To understand the utility of culture, we analysed our experience in a retrospective cohort of sequential patients aged 0–18 years admitted to hospital between 1 August 2021 and 31 March 2024, for workup for TB. Investigations were at the discretion of the managing clinicians; all patients had at least one stool submitted for mycobacterial culture. All specimens were submitted to the provincial reference mycobacteriology laboratory and cultured using liquid (BD BACTEC MGIT System) and solid (Lowenstein–Jensen) media according to local protocols derived from Ref. 5. All *Mycobacterium* spp. isolated were identified and reported. For stool specimens, an additional decontamination step occurred prior to routine processing of non-sterile specimens.^5^ Disseminated TB was defined as disease in two or more non-contiguous organs.^6^ Ethics approval was obtained from the Hospital for Sick Children’s Research Ethics Board (REB#1000080972). Written consent was obtained from the patients and/or guardians for the illustrative cases.

For the 19 patients identified, 35 individual stool specimens for culture were taken, of which 9 (26%) were positive in 8 (42%) patients. Four of the 19 patients had a clinical diagnosis; thus, 53% of the 15 patients with positive MTBC cultures from any site had a positive stool MTBC culture. Stool was the first culture-positive sample in 3/19 (16%) patients, and the only positive sample in 1 (5%) patient (Table). Only 2 stool samples were smear positive for AFB but one failed to grow mycobacteria on culture. More than 1 stool culture was sent in 9/19 (47%) patients. Six of the 19 (32%) patients had 3 stool specimens submitted: 4 had negative stool cultures; while for the 2 remaining patients, MTBC was isolated in 1/3 specimens. The small sample size did not permit robust statistical comparison: there was a trend to more positive cultures in those with disseminated TB (*P* = 0.064). Of the 9 patients with disseminated TB, 6 (75%) had a positive stool culture as did 2/9 patients (22%) with isolated pulmonary disease. The median time to culture positivity was 20 d for stool specimens, 10.5 d for respiratory specimens, and 14 d for all non-stool specimens (Table).

**Table.** Characteristics of patients admitted for TB workup from 1 August 2021 to 31 March 2024.

Patient characteristics	Cohort, n = 19
Age, years (median, IQR)	11 (1.7, 15)
Sex, male (n, %)	8 (42%)
Location of disease (n, %)^A^	
Disseminated	9 (47%)
Isolated pulmonary TB	9 (47%)
Isolated cervical lymphadenopathy	1 (5%)
Abdominal symptoms (n, %)	4 (21%)
Number of stools submitted/patient (median, IQR)	1 (1–3)
Culture results (per patient) (n, %)	
Culture negative for MTBC, presumed clinical TB	4 (21%)
Culture positive for MTBC, any site	15 (79%)
Stool culture positive:	8 (42%)
Stool = only positive culture	1 (5%)
First positive culture =stool culture	3 (16%)
Stool culture negative, other cultures positive	7 (37%)
NTM in stool culture^B^	5 (26%)
Time to positivity (TTP) (median, IQR)	
TTP for stool culture, days^C^	20 (18.8–26.3)
TTP for sputum and/or gastric aspirates, days	10.5 (9.3–14.3)
TTP for first non-stool^D^ culture, days	14 (10–17)

*P = 0.064; disseminated TB compared all with other cases. Statistics were conducted using R (version 4.4.3, R Core Team, Vienna, Austria, 2023); Fisher’s exact tests were used for categorical variables (expected small cell sizes).

IQR = interquartile range; MTBC = *Mycobacterium tuberculosis* complex; NTM = non-TB mycobacteria.

A Disseminated TB was defined as disease in two or more non-contiguous organs.

B NTM species isolated from stool were *M. gordonae*, *M. avium*, *M. fortuitum group*, and *M. chimaera*.

C 1/9 positive stool specimens excluded from TTP analysis due to collection post-treatment initiation.

D Non-stool culture includes sputum, BAL fluid, pleural fluid, gastric aspirates, CSF, urine, lung, and lymph node.

Of the 35 stool samples, 7 (20%) obtained from 5 patients (5/19, 26%) grew non-TB mycobacteria (NTM). Three of these patients had microbiologically confirmed TB disease. All 5 patients were treated for TB disease and not for NTM. No NTM-related disease was diagnosed over a minimum of 12 months of post-treatment follow-up and the NTM identified were thought to be contaminants. No other contaminating organisms were isolated from stool cultures.

Our experience suggests that stool culture has an important role in diagnosis and management in some cases of paediatric TB. There are few paediatric data for comparison. The yield of 53% for confirmed TB in our patients compares with 24% in a South African study of childhood pulmonary TB^7^ and 40% in adults and elderly patients with pulmonary disease in Japan.^8^ In those studies, the rates of molecular test positivity on stool were 36% and 68%, respectively. The high commensal bacterial load in stool may lead to high rates of contamination of specimens and result in an inability to perform MTBC phenotypic DSTs or the premature discontinuation of the culture. Contamination occurred in 41.5% of stool specimens in the above-cited South African study,^7^ significantly limiting the culture yield. In our patients, where an additional decontamination step was used, contamination rates were lower (20%) and limited to NTM. Although stool was the first positive culture in three patients, the major drawback was the generally longer time to positivity compared with other specimen types. The addition of molecular tests would likely significantly improve the utility of stool for TB diagnosis.

Limitations of our data include the absence of molecular tests as only smear-positive stools underwent PCR, and the small sample size in a low-burden setting. We did not investigate patients with milder disease managed in the outpatient setting. Concurrent molecular testing would likely have given much more rapid though incomplete information (without DSTs) and obviated invasive procedures in some of our patients. However, stool cultures were readily obtained without invasive procedures and provided us with microbiologic confirmation and DSTs that directly impacted clinical decision making for some patients. They may be especially useful where obtaining other cultures is difficult. Based on our experience, stool culture for MTBC is a potentially useful test in many cases of paediatric TB. Larger studies that include both molecular testing and culture under ideal conditions are needed to optimise the use of stool specimens for the diagnosis of TB in children and adolescents.
